# Acute HIV Infection in Pregnancy: The Case for Third Trimester Rescreening

**DOI:** 10.1155/2011/340817

**Published:** 2011-12-22

**Authors:** Jocelyn Wertz, Jason Cesario, Jennifer Sackrison, Sean Kim, Chi Dola

**Affiliations:** Department of Obstetrics and Gynecology, Tulane University School of Medicine, 1430 Tulane Avenue, SL-11, New Orleans, LA 70112, USA

## Abstract

Combination testing with anti-HIV Elisa and Western blot is both sensitive and specific for diagnosis of established HIV-1 infection but could not detect acute HIV infection (AHI). AHI is a time of extremely high viral load, which may correlate to increased risk of horizontal or vertical transmission. Thus, early identification of AHI could allow for interventions to decrease transmission. However, recognition of AHI can be challenging as symptoms could be absent or nonspecific, therefore, AHI is often not detected, particularly in pregnancy. We present a case report of AHI in a pregnant woman who presented with headache and fever. She tested negative for HIV in the first trimester and at time of AHI at 26 3/7 weeks by anti-HIV Elisa, but was diagnosed with AHI based on an HIV RNA viral load of 434,000 copies/mL. This report presents a case for improved awareness of AHI in pregnancy, and the need for repeat HIV testing in late pregnancy, and highlighted that early detection of AHI might be possible with adding HIV RNA testing at time of standard anti-HIV Elisa screening test in pregnancy. Novel laboratory approaches including pooling of sera for HIV RNA could reduce the cost of HIV RNA testing.

## 1. Case Report

A 23-year-old G1P0 at 26 weeks and 3 days gestation presented to the Emergency Department (ED) at a private hospital (henceforth referred to as hospital no. 1) complaining of a headache. Her past medical history was significant for chronic hypertension, depression, anxiety, and uterine fibroids. Her obstetric course had been complicated by intermittent bouts of abdominal pain since 4 weeks of gestation, for which she underwent a diagnostic laparoscopy significant only for a ruptured corpus luteum. She had been a patient of our teaching institution since early first trimester, at which time all prenatal labs were performed. The only pertinent findings were Rh-negative status and Trichomonas vaginalis infection. HIV Elisa test was negative. At approximately 20 weeks gestational age, the patient transferred her care from the teaching institution to a community physician. All prenatal labs were again repeated; other than an abnormal pap smear, all labs including HIV antibody test were negative. 

Social history obtained upon admission revealed her current partner, and father of the baby, was a 50-year-old man with a history of incarceration, residence in shelters for the homeless, and prior hospitalizations for respiratory infection that was suspicious for tuberculosis. Initial workup of the mother in the ED at hospital no. 1 revealed elevated liver enzymes in the range of 400–500's, leukopenia, and maternal fever. HIV Elisa was negative and HIV RNA viral load was ordered. The patient was admitted to the hospital. To evaluate the patients' headache, neurology and infectious disease consults were requested and a lumbar puncture was performed. VDRL and Cryptosporidium test were ordered and were negative. The working diagnosis at this time was disseminated infection with Herpes simplex virus (HSV). The infectious disease physician recommended intravenous acyclovir for 10 days. On antiviral therapy day number five, the patient requested transfer to another private hospital (henceforth identified as hospital no. 2) where her obstetrician had admitting privileges. Of note, HIV viral load was still pending at time of transfer.

Upon arrival at hospital no. 2, a Maternal-Fetal medicine specialist from our teaching institution was consulted to evaluate the presumed disseminated HSV infection. Social history in the transferring note received from hospital no. 1 stated that the patient's partner was currently hospitalized with renal failure and end-stage AIDS. The plan at this time was to continue intravenous Acyclovir for the presumed disseminated HSV infection while HIV workup was in progress. Repeating HIV rapid screen test, HIV RNA viral load, and CD4 and CD8 counts were done, a PPD test was placed, and workup was ordered for elevated liver enzymes. Consultations with infectious disease and hepatology were requested. WBC on admission to hospital no. 2 was 4.3; Hepatitis A, B, C serologies, Human Granulocytic Ehrlichiosis IgG and IgM, and Toxoplasmosis IgG and IGM were negative. A right upper quadrant ultrasound was performed and ruled out any pathology. The PPD was read as negative. Blood cultures were negative. Rapid screen HIV test was again negative.

 Several days after admission to hospital no. 2, laboratory results were received from hospital no. 1, which were significant for HIV viral load being greater than 500,000 copies/mL and CD4 count of 227 cell/mm^3^. Thus the patient received the new diagnosis of acute HIV infection. She was started on an antiretroviral regimen of Combivir 150 mg/300 mg 1 tablet twice daily and Viracept 625 mg 2 tablets twice daily.

 On hospital day 6, her liver function tests decreased to an AST of 191, ALT of 365, and an alkaline phosphatase of 111. HIV RNA viral load that was obtained on admission to hospital no. 2 returned as 434,000 copies/mL. CD4 count was 323.9 cell/mm^3^. Her symptoms of fever and headache resolved and she was later discharged home without further complications or findings. Of note, laboratory studies as an outpatient at approximately 3 weeks later demonstrated a CD4 count of 447 cell/mm^3^ and a viral load of 869,000 copies/mL. Antiretroviral therapy was continued as outpatient. At 33 weeks and 5 days gestational age, her CD4 count was 177 cell/mm^3^ and viral load was 128 copies/mL.

The patient presented to hospital no. 2 at 37 weeks and 2 days in early labor complicated by chronic hypertension with superimposed preeclampsia. She was admitted for delivery. Based on the last viral load being less than 1,000 copies/mL, she was allowed a trial of vaginal delivery. She received a loading dose of zidovudine at 2 mg/kg followed by maintenance dosage of 1 mg/kg throughout labor. She also received magnesium sulfate for seizure prophylaxis. The viral load during this admission was 74 copies/mL. She underwent a low transverse cesarean section secondary to arrest of dilation and gave birth to a 4 pound 11 ounce infant girl with Apgar scores of 8 and 9 at one and five minutes, respectively. The patient was discharged on postoperative day number three without complications. The infant was negative for HIV infection at four months of life.

## 2. Discussion

Estimates of the incidence of HIV infection in the United States range from 40,000 to 56,300 annually [1, 2]. At any time, up to 25% of HIV-infected individuals are unaware of their status [3]. Although the incidence of new HIV infection has held relatively steady throughout the 21st century, improvements in medical management of HIV patients has led to improved survival, thus the prevalence of the disease has increased [1]. There are now over 1,000,000 HIV-infected persons in the United States [1]. Although the total number of infected persons has increased, the incidence of new infections remains stable, which indicates a decline in transmission rates. However, while the incidence does not appear to have changed significantly over the last decade, the demographics of affected people have changed.

Women are making up a larger proportion of newly HIV-infected persons than ever before. Initially many HIV-infected women were intravenous drug users, however in recent years more women are acquiring HIV through heterosexual contact, many of whom do not have the previously identified risk factors for infection [4]. At this time, although there is more information regarding HIV infection in pregnant women, data is especially lacking in the detection and incidence of acute HIV infection in pregnancy. A study in North Carolina demonstrated the feasibility of identifying pregnant women with AHI, but due to the study design, it is impossible to infer AHI incidence among pregnant women [5].

Pregnant women have an elevated risk of HIV acquisition [6, 7]. Even when controlling for behavioral risk factors of women and their partners, pregnant women have twice the risk for infection when compared to breastfeeding women and nonpregnant, nonlactating women [6]. It has been proposed that hormonal changes associated with pregnancy as well as the impact of these hormones on the vaginal mucosa account for the increased susceptibility to infection [8–12]. These findings were further substantiated by another study that observed a 2-fold increased risk of HIV-1 acquisition during pregnancy [6]. Therefore, pregnancy is the time when women are at increased risk for AHI.

Acute HIV-1 infection involves dramatic alterations in viral load as well as the host's immune system which may increase the risk of both horizontal and vertical transmission during pregnancy [13]. Previous studies have demonstrated a 10-fold increased risk of horizontal transmission during AHI as compared to asymptomatic HIV infection [14]. This could be due to both the elevated viral load and/or the less frequent use of protective barrier methods as these persons are not aware of their infected status [4]. Because of the high viral load, an increase in vertical transmission is a valid concern if the woman delivers during the stage of acute infection. This is especially true because medical therapy and obstetrical interventions aimed at reducing transmission may not be offered to these women with AHI who are being misdiagnosed as HIV negative due to the inability to detect early HIV infection with current standard HIV antibody testing.

Vertical transmission continues to be of significant concern as the CDC reports HIV infection among infants to be 144–226 annually in the United States [15]. Lack of maternal HIV testing in early pregnancy and failure to receive appropriate prophylaxis were commonly cited as the reasons for these infected infants [15]. There is definite correlation between maternal HIV viral load and perinatal transmission [16]. As early HIV infection is associated with an increased viral load [17] and high viral load is directly related to an increased risk of vertical transmission [18], AHI at the time of delivery could result in increased perinatal transmission of HIV. There is little data to describe the risk of vertical transmission in delivery during the acute phase of HIV infection.

As acute HIV-1 infection may mimic other common viral infections, this disease may be misdiagnosed as in the patient presented. Furthermore, as there is low prevalence of AHI in pregnancy in the United States, obstetricians often rely too much on the negative ELISA antibody test to exclude the disease. Pregnant women who have initial HIV-1 screening done before antibody development may have undetected HIV infection [19]. It is known that the antibody to HIV infection does not develop until much later in the disease process. There are currently two methods for detecting AHI: HIV-1 RNA by reverse transcriptase polymerase chain reaction (HIV-1 RNA RT-PCR) and HIV-1 p24 antigen assay. Polymerase chain reaction (PCR) can be used to measure the quantitative plasma HIV-1 RNA level (viral load) by 11-12 days after infection, with a sensitivity close to 100 percent and a specificity from 95 to 98 percent [20, 21]. Detection of AHI by p24 antigen assay is possible as early as 14 to 15 days after infection. However, as p24 antigenemia is short-lived and declines as immune complexes form and anti-HIV antibody titers increase, its usefulness is limited [20]. Finally, antibody seroconversion is apparent from weeks 3 to 7 post-exposure only [18].

The diagnosis of acute HIV infection can be made with detection of a quantitative plasma HIV-1 RNA viral load greater than 50,000 copies/mL coincident with the absence of HIV antibodies [22]. Our laboratory uses the Cobas AmpliPrep TaqMan HIV test by Roche which quotes a detection rate at viral loads as low as 45 copies per mL [23]. Since ELISA antibody screening tests can be falsely negative in early infections, should these screening tests in pregnancy be followed by reflex RNA viral load testing? This question becomes especially important because of the ramifications of vertical transmission. While p24 antigen assay and HIV-1 RNA RT-PCR are extremely effective means of HIV-1 detection, their cost prohibits its use as universal screening tools. As the benefits of detecting AHI in pregnancy appear to be great, we might consider using HIV-1 RNA RT-PCR following the initial screening test. To decrease costs, a less expensive screening method with satisfactory rates of AHI detection utilizing pooled serum for HIV-1 RNA screening could be considered [24–26].

When compared to the p24 antigen assay, several studies demonstrate increased sensitivity as well as lower cost with pooled serum HIV-1 RNA screening [25, 26]. PCR of pooled sera has comparable sensitivity to single sample PCR, but with the added benefits of increased test efficiency and decreased cost of diagnostic screening [25]. In a study in India, Quinn et al. describe a multistage system of serum pooling and testing for HIV-1 RNA via reverse-transcriptase polymerase chain reaction that was more sensitive for identifying women with AHI when compared to p24 antigen detection [26]. It also demonstrated a decreased number of tests carried out by 78% while decreasing the cost of detecting individuals with new infections by 34.4% [26]. Furthermore, this system becomes more cost-effective as prevalence of HIV infection in the population declines [26]. As such, implementation of this system in the United States may lessen the financial burden of screening.

Using the serum pooling method, Pilcher et al. estimated cost of RNA assay at $2 per specimen [27]. Comparatively, the financial burden of caring for a child with perinatally acquired HIV infection can be extremely high. Wilson et al. predicted the cost of treatment in the HAART era to be $1,820 per month in 2007 dollars, with a cost of treatment over 15 years of $181,436 [28]. In 2009, there were 4,131,019 births in the United States [29]. If all of these births were singletons carried long enough for the mothers to have received repeat third trimester HIV screening, we can make a gross estimate of the additional cost of nationwide screening with serum pooling method for RNA assay (at $2 per specimen) to be $8.26 million. The cost of 15 years of care of 144 HIV-infected neonates, the low end of the number of cases of vertical transmission in the US each year, is $26.13 million. This results in an estimated saving of $17.87 million. While these figures are gross estimates at best, even these rudimentary calculations demonstrate the potential for substantial savings if third trimester screenings were implemented.

In reviewing the literature, we found an important case report from Steele [30]. He describes three cases of infants born to mothers with negative HIV screening tests in early pregnancy. HIV infection in these three women was identified shortly after delivery. Two scenarios are plausible: these women were screened during the window period of infection or they became infected later in pregnancy. Two of the three infants developed HIV infection [30]. This is supported by one study of 407 HIV-positive mothers which detected eight seroconversions following negative HIV-1 testing during or immediately prior to pregnancy, with perinatal HIV transmission following three of these eight sero-conversions [31].

This case report from Steele highlights the importance of rescreening for HIV infection during pregnancy. Screening with reflex HIV RNA PCR testing and rescreening in late pregnancy may have detected some if not all of these cases. Following CDC guidelines, these women were considered to be low risk and were therefor not retested later in pregnancy [15, 32]. Again, these women were found to be infected with HIV after delivery and two of three infants acquired HIV infection. ACOG guidelines currently recommend first trimester care to include routine opt-out HIV-1 screening [33]. High-risk patients, including those residing in 20 states with high HIV incidence, those who receive prenatal care at facilities with an HIV incidence of at least 1 per 1000 women screened, those who exhibit signs or symptoms of acute HIV infection, and those with high-risk behaviors, qualify for retesting [15, 32]. While the patient presented in this report would have been retested for HIV in the third trimester but most likely with the current standard HIV Elisa screening test, it is difficult to know if she would have been identified if the timing of the test was within the window period (3–7 weeks after exposure) when antibody was not developed yet. Given the continued cases of vertical transmission despite HIV screening in pregnancy, the question arises: are the current US screening tests and guidelines adequate?

Reduced perinatal transmission may be achieved with repeat HIV testing in late pregnancy as well as during labor and delivery, as recommended by Patterson et al. [5]. However, while repeat third trimester testing would undoubtedly identify some newly infected individuals, others could still be in the window phase. For this reason, further recommendations of reflex RNA testing for antibody-negative women to detect acute HIV infection should be considered [5]. Although this paper focuses on testing in the United States, several studies have demonstrated the feasibility of reflex RNA testing in developing countries [24–26].

The management of HIV in nonpregnant persons has been well studied. There are existing guidelines of when and how to initiate treatment [18]. However, the management of acute HIV infection is a less well-studied entity. Current studies indicate that HAART therapy in acute HIV infection decreases viremia [34] and thus in theory may reduce vertical transmission. Unfortunately, there are few studies describing the impact of AHI in pregnancy (with its high viral load) and perinatal outcomes. This could be an area of future research.

## 3. Conclusion

This case study demonstrates the continuing concerns regarding current HIV screening recommendations during pregnancy and the difficulty of recognizing and diagnosing acute HIV infection. Obstetricians must be able to recognize acute HIV infection and should understand how to make the diagnosis. More research must be done, as there is a paucity of data describing AHI in pregnancy and its impact on perinatal outcomes.

This case study reinforces the importance of understanding the timeline of HIV infection from first exposure to development of anti-HIV-1 antibodies and the implications for early detection of infection. Due to the seronegative period of acute HIV infection, we support recommendations for reflex viral load testing on all HIV-1 screening in pregnancy [5]. The potential increased risk of acquiring HIV infection during pregnancy due to hormonal change, and cases where HIV-1 negative women tested in early pregnancy delivered babies who tested positive shortly after birth also encourage us to recommend third trimester re-screening of HIV-1 in all pregnant women regardless of their risk factors [6, 19]. Additional testing in late pregnancy would allow women and their unborn children to benefit from interventions, which reduces vertical transmission of HIV-1.

It is thought that the public health benefits of identifying HIV-infected patients during acute infection will outweigh the cost of viral load testing in areas with high HIV transmission [35]. Further cost-benefit studies to assess the application of repeat testing in the United States would be illuminating. The increased costs of assessing viral load in addition to rapid screens may be reduced by using pooled sera methods of detection. As pointed out by Steele [30] unless the CDC revises its guidelines to include repeat HIV screening in late pregnancy and allow for reflex viral testing in all pregnancies, the costs for these tests might not be covered by insurance companies [30]. We hope that changes in guidelines will be initiated soon to effectively detect AHI in pregnant women and prevent mother-to-child transmission of the disease whenever possible.
